# Livédo réticulaire post vaccin anti COVID-19: à propos d’un cas

**DOI:** 10.11604/pamj.2024.49.9.31779

**Published:** 2024-09-09

**Authors:** Meryem Ouhaddach, Mouna Zahlane, Lamiaâ Essaadouni

**Affiliations:** 1 Service de Médecine Interne, Hôpital Arrazi CHU Mohamed VI, Marrakech, Maroc

**Keywords:** Livédo réticulaire, vaccination, effet indésirable, cas clinique, Livedo reticularis, vaccination, adverse reaction

## Abstract

L'apparition du syndrome respiratoire aigu sévère coronavirus 2 a rendu crucial le développement de vaccins sûrs, dont trois principaux types existent à ce jour. Bien qu'ils aient montré une excellente sécurité, ils ont provoqué divers effets indésirables. Nous présentons un cas rare de livédo réticulaire post-vaccinal chez une femme de 54 ans, apparu 24 heures après la vaccination, accompagné de troubles respiratoires, digestifs et neurologiques, et d'une altération de l'état général. Les examens ont révélé des lésions cutanées compatibles avec un livédo réticulaire, un syndrome inflammatoire léger, et une hypercholestérolémie. L´imagerie était sans particularité. Un bilan immunologique a montré un anticoagulant lupique positif. La patiente a été traitée symptomatiquement, avec une amélioration des symptômes neurologiques et articulaires, et une légère régression des éruptions cutanées. Après trois mois, le test de l'anticoagulant lupique est resté positif, confirmant un syndrome des anticorps antiphospholipides (SAPL) post-vaccinal. L´association du livédo réticulaire au vaccin contre la COVID-19 ne doit pas être sous-estimée, et son degré de gravité reste à déterminer. Il est nécessaire de collecter davantage de données et de cas pour une analyse plus approfondie et détaillée.

## Abstract (en)

The emergence of severe acute respiratory syndrome coronavirus 2 has made it crucial to develop safe vaccines. Three main types of vaccines are currently available. Although they have proven to be very secure, they have caused various adverse effects. We here report a rare case of livedo reticularis following the administration of COVID-19 vaccine in a 54-year-old woman. It occurred 24 hours after vaccination, accompanied by respiratory, digestive, and neurological disorders and deterioration in general condition. Examinations revealed skin lesions compatible with livedo reticularis, mild inflammatory syndrome and hypercholesterolemia. Imaging showed no abnormalities. Immunological tests were positive for lupus anticoagulant. The patient received symptomatic treatment, with improvement in neurological and joint symptoms and a slight regression of skin rashes. After three months, the lupus anticoagulant test remained positive, confirming post-vaccination antiphospholipid syndrome (APS). The association of livedo reticularis with COVID-19 vaccine should not be underestimated and its degree of severity remains to be determined. More data and cases need to be collected for a more in-depth and detailed analysis.

## Introduction

Au cours du temps, la vaccination a joué un rôle crucial dans l'amélioration de la santé. Elle a permis d´éradiquer les affections les plus mortelles du monde notamment la variole et la peste bovine. En revanche, l´inquiétude demeure dans le fait que ces vaccins peuvent induire certains évènements secondaires. Jusqu´en fin mars 2021, la pandémie de COVID-19 avait touché 219 pays et territoires avec infection de 125 millions et décès de plus de 2.7 millions de personnes; par conséquent, l´impact mondial du syndrome respiratoire aigu sévère coronavirus 2 (SARS-CoV-2) a rendu le développement de vaccins efficaces et sûrs capital pour cette nouvelle souche [1]. Jusqu'à présent, il existe trois principaux types de vaccins COVID-19 utilisés dans le monde: vaccins à acide ribonucléique messager (ARNm): BNT162b2 (Pfizer-BioNTech, New York, New York) et ARNm-1273 (Moderna, Inc., Cambridge, Massachusetts), vaccins vectoriels adénoviraux: ChAdOx1 nCoV-19 (AstraZeneca-Oxford), Gam-COVID-Vac (Gamaleya National Center of Epidemiology and Microbiology), A26.COV2.S (Janssen Pharmaceuticals, Inc) et Ad5-nCoV (CanSinoBIO) et vaccins à virus entiers inactivés: BBIBP-CorV (Sinofarm) et CoronaVac (Sinovac Life Sciences). Bien que les trois vaccins contre la COVID-19 aient démontré une efficacité et un profil de sécurité globalement satisfaisants, ils ont également été associés à un large éventail d'effets indésirables. Cependant, à notre connaissance, le livédo réticulaire n'a pas encore été décrit dans la littérature comme une réaction post-vaccinale [2]. Par conséquent, nous présentons ici un cas de livédo réticulaire post-vaccinal chez une femme de 54 ans.

## Patient et observation

**Informations du patient:** une femme âgée de 54 ans, ayant comme antécédant un rhumatisme articulaire aigu (RAA) traité à l´enfance et un diabète de type 2 sous Glimépiride bien équilibré. N´ayant eu aucune exposition connue au SARS-CoV-2, elle s´est présentée dans notre formation deux mois après l´administration d´une première dose de vaccin ChAdOx1-S/nCoV-19 dans un tableau fait d´un essoufflement, d´une toux productive et d´expectorations hémoptoiques, des troubles digestifs à type de diarrhées liquidiennes et de vomissements bilieux ainsi que d´arthro-myalgies et d´asthénie profonde. Quelques jours après, l´aggravation a été marquée par l´apparition de froideur des extrémités avec dysesthésie à type de brûlures et de fourmillements des membres. La patiente rapporte que cette symptomatologie a été initiée 24 heures après le vaccin.

**Figure 1:**
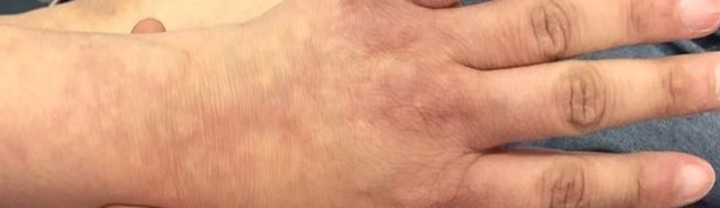
lésions de livédo réticulaire au niveau des membres supérieurs

**Figure 2:**
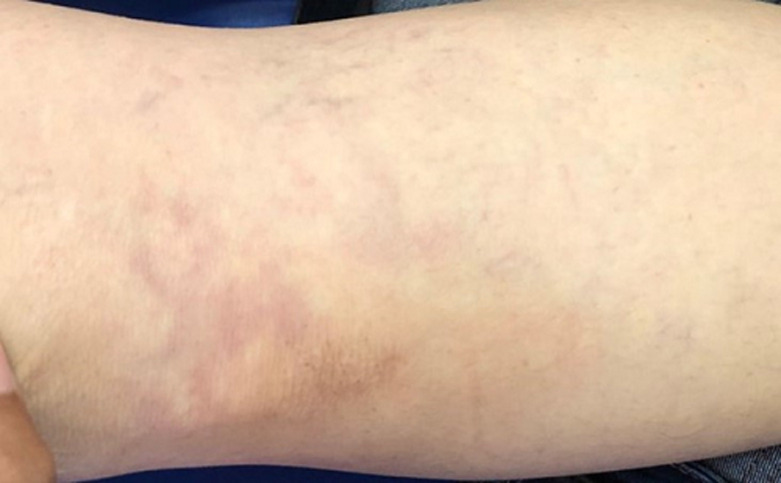
lésions de livédo réticulaire au niveau du membre inférieur gauche

**Résultats cliniques:** l´examen neurologique a objectivé des forces musculaires diminués à 3/5 au niveau des membres inférieurs avec hypoesthésie au niveau des plantes des pieds. L´examen cutanéo-muqueux a révélé des plaques érythémateuses de dentelle réticulaire bien délimitées compatible avec un livédo réticulaire diffus au niveau des quatre membres (Figure 1, Figure 2). Le reste de l´examen somatique a été sans particularités.

**Démarche diagnostique:** le bilan biologique a objectivé une anémie hypochrome microcytaire ferriprive (Hb: 9.8g/dl, VGM: 79 fl, TCMH: 23 pg, ferritinémie: 15mg/l), les autres lignées sanguines étant sans anomalies. La protéine C-réactive (CRP) était à 30mg/l et le bilan lipidique recontrôlé à deux reprises était perturbé (Cholestérol total à 2.5g/l, Triglycérides à 1.4g/l, HDL cholestérol à 0.45g/l, LDL cholestérol à 1.7g/l). Le diabète était équilibré (Hb glyquée à 7%). Les D-dimères était négatifs (200 ng/ml). Le reste de bilan notamment le bilan hépatique, rénal, le bilan d´hémostase, le fibrinogène, les sérologies (hépatite B/C, syphilis), le complément ainsi que le bilan immunologique (AAN, Ac anti-DNA, Ac anti-cardiolipines, Ac anti-B2 glycoprotéine, Ac anti-phospholipides, Anti-MPO, Anti-PR3) était négatif. La réaction en chaîne de la polymérase nasopharyngée (PCR) couplée à la sérologie COVID-19 (IgM et IgG) ont été également réalisées n´objectivant que des IgG élevés.

Sur le plan radiologique, un angioscanner thoracique réalisé à la recherche d´éventuelle embolie pulmonaire était normal. L´échographie cardiaque transthoracique n´as pas mis en évidence d´anomalies. L´échographie doppler artérioveineuse des quatre membres a révélé une légère infiltration athéromateuse des membres inférieurs sans retentissement d´amont. Devant le tableau neurologique, l´électroneuromyogramme fait était normal et l´angio-IRM cérébro-médullaire montrait des discopathies dégénératives C5-C6 et L4-L5 non conflictuelles. Dans le cadre de recherche d´éventuelle vascularite, des biopsies cutanées de quatre millimètres du poignet et des cuisses ont été réalisées objectivant une dermatite superficielle chronique non spécifique.

**Intervention thérapeutique et suivi:** sur le plan thérapeutique, la patiente a reçu un traitement symptomatique à base de prégabaline (75mg x 3/jr), antiagrégant plaquettaire (acide acétylsalicylique à dose de 100mg/jr) et des statines (20mg/jr). L´évolution a été marquée par la résolution des symptômes initiaux avec régression des douleur neurologiques et articulaires ainsi que des manifestations cutanées après 3 mois de traitement. Devant la négativité de tout le bilan, un livédo post vaccinal a été retenu.

**Perspective du patient:** pendant l'hospitalisation et à la sortie, la patiente était ravie des soins.

**Consentement du patient:** le consentement éclairé a été obtenu de la part de la patiente pour que nous utilisions le cas.

## Discussion

L´efficacité des vaccins COVID-19 a été objectivée dans plusieurs études, de plus, leur administration a augmenté. L´immunogénicité chez les personnes ayant déjà été infectées étant donnée qu´ils avaient des titres d'anticorps plus élevés que celles sans infection [3]. Cela, reste à tester, pourrait expliquer le large spectre d´effets indésirables provoqués. Une étude observationnelle prospective a été faite au Royaume Uni afin d´évaluer les différents effets secondaires systémiques aussi bien que locaux observés dans les huit jours suivant la vaccination (BNT162b2, Vaccin ChAdOx1 nCoV-19); il a été constaté que la fatigue et les céphalées étaient les symptômes généraux les plus fréquemment rapportés dans les 24 premières heures après la vaccination suivi de frissons, diarrhées, arhtromyalgies et nausées. Quant aux effets secondaires locaux, la douleur locale au site d´injection était aperçue au premier rang tandis que les réactions allergiques telles que les brûlures et les éruptions cutanées ont été signalées que par une minorité [4].

La majorité des réactions secondaires qui ont survenue au cours des essais du vaccin ChAdOx1 nCoV-19 étaient de gravité légère à modérée, et elles étaient toutes spontanément résolutives. Les lésions rapportées étaient principalement des cas de zona et un seul cas de rosacée. Ceci peut s´expliquer par le fait que c´est un vaccin qui utilise un adénoviral non humain lui permettant ainsi d´éviter le risque d'immunité préexistante contre le vecteur et d'augmenter son efficacité [5]. Les réactions inflammatoires retardées ont été rarement notées; le seul cas connu a été décrit chez une femme de 68 ans avec un antécédent de sclérodermie localisée par l'apparition d'une éruption papulo-érythémateuse très prurigineuse diffuses. C´est une symptomatologie qui s´est développée trois jours après la première dose du vaccin [6].

Quant aux événements thromboemboliques provoqués par ce vaccin, il a été déclaré que la présence de caillots sanguins avec une thrombopénie est un effet secondaire grave mais très rare. C´est le cas de la thrombopénie immunitaire pro-thrombotique induite par le vaccin (VIPIT). C´est une réaction qui peut se présenter sur la peau sous forme d´éruption pétéchiale, d´érythème ou d´œdème des extrémités, comme elle peut être également systémique se manifestant essentiellement par des céphalées persistantes et sévères, des symptômes neurologiques focaux, des crises convulsives, une vision floue, un essoufflement et des douleurs thoraciques ou abdominales, s'avérant être un signe précieux d'une maladie potentiellement mortelle ne devrait pas être sous diagnostiquée [7].

Dans notre cas, la patiente a rapporté une symptomatologie proche des données de la littérature en plus d´un livédo réticulaire (LR) ce qui est une manifestation non décrite à la limite de nos connaissances. L´état de santé de la patiente avant l´administration du vaccin était bon avec un bilan totalement négatif et un manque de pharmacothérapie nouvellement initiée. Ces informations ont renforcé l'association entre livédo réticulaire et le vaccin. Il faut noter que même dans le cas d´un LR pathogène, les modifications vasculaires microscopiques sont globalement difficiles à détecter, car l'inflammation peut être distribuée de manière segmentaire ou il peut y avoir une inflammation de bas grade, dans laquelle les vaisseaux perméables ne discernent pas de vascularite [8].

Dans cette situation, une biopsie incisionnelle s´avère alors nécessaire afin d´augmenter le rendement diagnostique, permettant une évaluation plus approfondie des vaisseaux cutanés, mais cela n´a pas pu été réalisée chez notre patiente. Il a été rapporté quelques cas l´associant avec le SARS-CoV-2 [9]. Etant un virus avec un tropisme de grande envergure, il a été suspecté qu´il provoquait autant de dommages dans les cellules endothéliales. Une étude a confirmé cette hypothèse par la mise en évidence de la protéine de pointe du SARS-CoV-2 directement dans le cytoplasme des cellules endothéliales entraînant ainsi des modifications vasculaires aboutissant à une altération de la coagulation et de l'homéostasie vasculaire et par conséquent provoquer un LR (26) [10]. Cela ne peut pas être le cas de notre patiente vu qu´elle n´avait pas présenté d´infection antérieure par le COVID documenté par la négativation de sa sérologie (IgM) et de sa PCR COVID-19.

## Conclusion

Il est important de penser devant une vaccination anti-Covid spécifiquement le vaccin ChAdOx1 nCoV-19 aux manifestations cutanées y compris le livédo réticulaire. Son association ne doit pas être sous-estimée et son degré de gravité reste encore à le déterminer. Désavantage de données et de cas doivent être collectées avec une analyse plus approfondie et plus détaillée.
